# Spatio-temporal GAMLSS modeling of the incidence of schistosomiasis in the central region of the State of Minas Gerais, Brazil

**DOI:** 10.1590/0102-311XEN068822

**Published:** 2023-06-26

**Authors:** Denismar Alves Nogueira, Thelma Sáfadi, Renato Ribeiro de Lima, Angélica Sousa da Mata, Miriam Monteiro de Castro Graciano, Joziana Muniz de Paiva Barçante, Thales Augusto Barçante, Stela Márcia Pereira Dourado

**Affiliations:** 1 Universidade Federal de Alfenas, Alfenas, Brasil.; 2 Universidade Federal de Lavras, Lavras, Brasil.

**Keywords:** Residence Characteristics, Biomphalaria, Regression Analysis, Secondary Data Analysis, Distribuição Espacial, Biomphalaria, Análise de Regressão, Análise de Dados Secundários, Distribución Espacial, Biomphalaria, Análisis de Regresión, Análisis de Datos Secundarios

## Abstract

In Brazil, millions of people live in areas with risk of schistosomiasis, a neglected chronic disease with high morbidity. The *Schistosoma mansoni* helminth is present in all macroregions of Brazil, including the State of Minas Gerais, one of the most endemic states. For this reason, the identification of potential foci is essential to support educational and prophylactic public policies to control this disease. This study aims to model schistosomiasis data based on spatial and temporal aspects and assess the importance of some exogenous socioeconomic variables and the presence of the main *Biomphalaria* species. Considering that, when working with incident cases, a discrete count variable requires an appropriate modeling, the GAMLSS modeling was chosen since it jointly considers a more appropriate distribution for the response variable due to zero inflation and spatial heteroscedasticity. Several municipalities presented high incidence values from 2010 to 2012, and a downward trend was observed until 2020. We also noticed that the distribution of incidence behaves differently in space and time. Municipalities with dams presented risk 2.25 times higher than municipalities without dams. The presence of *B. glabrata* was associated with the risk of schistosomiasis. On the other hand, the presence of *B. straminea* represented a lower risk of the disease. Thus, the control and monitoring of *B. glabrata* snails is essential to control and eliminate schistosomiasis; and the GAMLSS model was effective in the treatment and modeling of spatio-temporal data.

## Resumo

No Brasil, milhões de pessoas vivem em áreas de risco para a esquistossomose, uma doença negligenciada, de caráter crônico e com elevada morbidade. O helminto *Schistosoma mansoni* está presente em todas as macrorregiões, incluindo o Estado de Minas Gerais, um dos mais endêmicos. Por essa razão, a identificação de potenciais focos é fundamental para subsidiar políticas públicas de cunho educativo e profilático no controle desse desfecho. Nesse contexto, o objetivo do trabalho consiste em modelar dados de esquistossomose em relação aos aspectos espaciais e temporais, além de avaliar a importância de algumas variáveis exógenas socioeconômicas e a presença das principais espécies de *Biomphalaria*. Como trabalhar com casos incidentes, uma variável discreta de contagem, exige uma modelagem apropriada, foi escolhida a modelagem GAMLSS por considerar conjuntamente uma distribuição mais adequada à variável resposta devido à inflação de zeros e à heterocedasticidade espacial. Verificaram-se valores elevados de incidência em diversos municípios de 2010 a 2012 e uma tendência de queda até 2020. Também foi identificado que a distribuição da incidência se comporta de maneira diferente no espaço e no tempo. Municípios com barragem apresentaram risco 2,25 vezes maior do que os que não a continham. A presença de *B. glabrata* foi relacionada ao risco de ocorrência da doença. Por outro lado, a presença de *B. straminea* refletiu em menor risco de ocorrência da esquistossomose. Conclui-se que o controle e o acompanhamento dos caramujos da *B. glabrata* podem ser fundamentais para a contenção e a eliminação da esquistossomose e o modelo GAMLSS foi eficaz para tratamento e modelagem de dados espaçotemporais.

## Resumen

En Brasil, millones de personas viven en áreas de riesgo de esquistosomiasis, una enfermedad crónica desatendida y con alta morbilidad. El helminto *Schistosoma mansoni* está presente en todas las macrorregiones, incluido el Estado de Minas Gerais, uno de los más endémicos del país. Por ello, la identificación de potenciales brotes es fundamental para promover políticas públicas de carácter educativo y profiláctico en el control de este desenlace. En este contexto, el objetivo de este trabajo es modelar datos sobre esquistosomiasis con respecto a aspectos espaciotemporales, además de evaluar la importancia de algunas variables socioeconómicas exógenas y la presencia de las principales especies de *Biomphalaria*. Dado que en el trabajo con casos incidentes una variable de conteo discreta requiere un adecuado modelado, se eligió el modelo GAMLSS, ya que en conjunto considera una distribución más adecuada para la variable de respuesta debido a la inflación de ceros y la heterocedasticidad espacial. Se encontraron valores de alta incidencia en varios municipios en el periodo evaluado de 2010 a 2012 y una tendencia a descenso hasta 2020. También se verificó que existe una distribución de incidencia de manera diferente en el espacio y el tiempo. Los municipios con represas presentaban 2,25 veces más riesgo que los que no las tenían. La presencia de *B. glabrata* estuvo relacionada con el riesgo de la enfermedad. Por otro lado, la presencia de *B. straminea* ocasionaba un menor riesgo de padecer la enfermedad. Se concluye que el control y seguimiento de caracoles *B. glabrata* puede ser fundamental para el control y eliminación de la esquistosomiasis y que el modelo GAMLSS resultó ser efectivo para el tratamiento y modelado de datos espaciotemporales.

## Introduction

Schistosomiasis is a serious and neglected tropical disease caused by parasites of the genus *Schistosoma* and affects countries worldwide, leading to the death of up to 280,000 people per year ^1^. According to the World Health Organization (WHO) ^2^, in 2019, more than 236 million people were affected by this disease. In Brazil, it is estimated that more than 25 million people live in risk areas; the parasite is present in all macro-regions of the country, with the State of Minas Gerais, in Southeast Region, being one of the most endemic states.

Several studies have reported that the infection occurs during agricultural and recreational activities and due to exposure to water contaminated with cercaria and, therefore, associated with the peripheries of cities where the lack of infrastructure and environments without sewage treatment is more common ^3^ ^,^ ^4^. In Brazil, the endemic form is caused by the helminth *S. mansoni*, responsible for the hepatosplenic form of schistosomiasis, although there are reports of some cases from other species ^5^. According to Souza et al. ^6^, 523 municipalities in Minas Gerais, or 61% of the total number of municipalities in the state, are considered endemic due to their high infection rates and because they contain a wide geographic distribution of snail species (*Biomphalaria* spp.), which is an intermediate host of the parasite. According to Massara et al. ^7^, the species *B. glabrata*, *B. tenagophila*, and *B. straminea*, which may or may not be infected with *S. mansoni*, are commonly found in Minas Gerais. Some disease control programs have been successfully implemented, and improvements in the health system with mass drug treatment have decreased the number of cases; nevertheless, the disease persists, maintaining its global relevance ^8^. Moreover, according to the WHO, drug treatment in some regions has not shown satisfactory results, and a possible control of the intermediate host may be an important measure in the control of the disease ^9^.

Currently, control depends on municipal public policies ^10^. Over 500 deaths have been verified in Brazil in the last 15 years. According to Silva da Paz ^5^ and Simões et al. ^8^, many cases have occurred in older adults (> 60 years), increasing the risk associated with chronic non-infectious diseases.

The State of Minas Gerais has been considered endemic for some years and studies, such as the one by Cardoso et al. ^11^, which evaluated the spatial and temporal aspect of deaths due to schistosomiasis, highlight the Vale do Aço, central, and northeastern regions of the state as the most worrisome. The identification of potential foci is essential for the planning of educational and prophylactic public policies for the control of schistosomiasis, especially in historically endemic regions, since this disease causes relevant impacts to those infected and, if untreated, can result in substantial morbidity or death ^12^ ^,^ ^13^.

Thus, the monitoring of schistosomiasis incidence and its associated variables is extremely important. Different forms of analysis and modeling have been used to approach this topic; the spatio-temporal analysis methodologies, however, have gained strength in recent years for their adequacy and efficiency in evaluating the effects of the disease considering space and time. Paz et al. ^14^ used this type of approach to evaluate the behavior of schistosomiasis in the Northeast Region of Brazil, enabling a better understanding of the problem and supporting public health decisions.

Notably, working with incidental cases, which is a discrete variable, requires a modeling of generalized methods since it allows for a more adequate distribution to the data. Unlike the generalized linear models (GLM) ^15^ and generalized additive models (GAM) ^16^, the generalized additive models for location, scale, and shape (GAMLSS) ^17^ are more flexible since they allow for the use of a wide variety of probability distributions for the dependent variable. This flexibility allows the modeling of the different distribution parameters, such as mean, variance, asymmetry, and kurtosis. Thus, one can associate different linear predictors to the different parameters of a given distribution, considering different binding functions. The terms of the linear predictors may contain parametric and smoothing (nonparametric) functions and may be of fixed or random effects. With the use of GAMLSS modeling we are able to assess not only how the mean of the dependent variable is influenced by the explanatory variables, but also how the variance and the other shape parameters of the distribution of the random (dependent) variable are influenced by the same or different explanatory variables.

This study aims to model schistosomiasis data in relation to spatial and temporal aspects, in addition to evaluating the risk and importance of some socioeconomic exogenous variables considering the presence of the main species of *Biomphalaria* in the central region of the State of Minas Gerais.

## Methodology

This ecological study uses secondary data from the public domain of the Brazilian Health Informatics Departments (DATASUS) regarding the occurrence of schistosomiasis in the State of Minas Gerais, from the Brazilian Information System for Notifiable Diseases (SINAN). The area in focus refers to the residents in the central region of the state, comprising the municipalities that border the Paraopeba River and its primary and secondary neighboring municipalities. The outcome variable refers to the sum of cases in the 103 municipalities, from 2007 to 2020, available in the SINAN database. The population of the region is of 6,182,086 inhabitants, according to the 2010 census conducted by the Brazilian Institute of Geography and Statistics (IBGE) ^18^. The exogenous variables include the M-HDI (Municipal Human Development Index) ^18^; flood events in the reports of emergency situation (Brazilian National Water Resources Information System - SNIRH of the Brazilian National Water and Sanitation Agency - ANA) ^19^; percentage of the population who does not have treated water and sewage (ANA) ^19^; percentage of the population with inadequate sanitation; rate of illiteracy among the population; percentage of the population with open sewage; percentage of the population living on 1/4 of the minimum wage per capita; rate of households without a bathroom (2010 IBGE census) ^18^; proximity to the Paraopeba River (municipalities that are in direct contact with the banks of the river; second-order and third-order neighbor municipalities); presence of mining residue dams in the municipality (Brazilian National Mining Agency - ANM) ^20^; presence of snails from the three intermediate host species, according to the report issued by the technical advisory committee of the schistosomiasis program of the Brazilian Ministry of Health (2008) ^21^; and number of inhabitants (offset). Other variables such as year, municipalities, and first and second order lags (*Y*_*i-1*_ for one year before and *Y*_*i-2*_ for two years before within each municipality) were also considered in the modeling to assess spatial and temporal dependence. The study uses secondary sources in the public domain and therefore respects ethical principles.

We chose to use GAMLSS modeling, as proposed by Rigby & Stasinopoulos ^17^. This methodology allows working with different probability distributions (not just of the exponential family), or with a mixture thereof, in the adjustment of regression and modeling of space and time. According to Rigby & Stasinopoulos ^17^, the GAMLSS regression for n independent observations, which is not the case of our study, are structured in the random effects and in the covariance matrix, leading to the understanding that the joint distribution will be conditional to these past values. It is normal to assume that the distribution or density function can present four (*k =* 1,…, 4; or more) parameters - location (*μ*), scale (*σ*), form (*ν* and *τ*) - when writing the bond function *g*_*k*_ (.) as a known monotone, relating *θ*_*k*_ *= (θ*_*k1*_ *, ..., θ*_*kn*_ *)*to the predictor variables and random effects through:

$$g_{k}\left(\theta_{k}\right)=X_{k}\beta_{k}+\sum_{j=1}^{J_{k}}h_{jk}\left(x_{jk}\right)$$

in which *X*_*k*_ is an array of covariates, *β*_*k*_ is the vector with the parameters associated with the covariates of each of the *k* parameters; *h*_*jk*_ is a nonparametric smoothing function applied to some of the continuous exogenous variables, considering that *J*_*k*_ are the functions applied to each of the parameters. The additive part, on the right side of the equation, can be replaced by *Σ Z*_*jk*_ *γ*_*jk*_ (or added) when the random effects are included in the model, in which $\gamma_{jk}∼N_{qjk}\left(0,\lambda_{jk}^{-1}G_{jk}^{-1}\right)$ with $G_{jk}^{-1}$ is the inverse (generalized) of the symmetric matrix (*q*_*ij*_ x *q*_*ij*_ ), considering that qij is the number of random variables, which in this study represents the number of municipalities or areas. According to De Bastiani et al. ^22^, random effects can be characterized as a Gaussian Markov random field (GMRF) and, therefore, it is possible to assume the matrix G as a weight or neighborhood structure in spatial modeling, thus obtaining the IAR (intrinsic autoregressive) models, coming from the CAR (conditional autoregressive) models, since G is singular.

The estimation of the parameters of the model is given by penalized maximum likelihood, in which the *β*, *γ* are estimated. The *λ* (hyperparameters present in the likelihood penalty function) can be fixed or estimated by the penalized quasi-likelihood. Stasinopoulos et al. ^23^ detail the processes and algorithms for estimation. For the analysis, we used the family of GAMLSS R packages (http://www.r-project.org) and others such as *mgcv* ^24^ and *spdep* ^25^.

To define the variables that make up the model, as recommended by De Bastiani et al. ^22^, the stepwise procedure and the generalized Akaike information criterion (GAIC) were considered, which can be reduced to both criteria Akaike information criterion (AIC) ^26^ (when the penalty is equal to 2) and bayesian information criterion (BIC) ^27^ (when the penalty is ln(n)). The selection of the best model was performed after assessing the quality of adjustment by means of normalized randomized quantile residuals ^28^, evaluated by Q ^29^ statistics and with the confirmation of independence with the use of correlograms.

To evaluate the distribution that best adheres to the data, a study was conducted with several discrete distributions that could encompass the excess of zeros and the long tail. Distributions such as zero-inflated poisson (ZIP), zero-inflated negative binomial (ZINBI), poisson inverse gaussian (PIG), and Sichel distribution were performed in addition to the variations of these distributions adjusted or altered to model the excess of zeros. A total of 16 distributions were evaluated and the one that best adhered to it was chosen, according to the GAIC criterion. Further details on the distributions can be found in Rigby et al. ^30^.

To avoid collinearity problems, exogenous variables that presented a correlation higher than |0.6| were eliminated; variables such as illiteracy rate, percentage of the population with open sewage, percentage of the population living with 1/4 of the minimum wage per capita, use of cesspools were represented by the M-HDI. The variables proximity (1 - closer, 2, and 3), presence of dam (0.1), and presence of the snail of the three species (*B. glabrata*, *B. straminea*, and *B. tenagophila*) (0.1) were considered as factors. As offset, we used the logarithm of the total population of each municipality and considered it constant in the study period.

### Map and region of interest

With the development of digital map creation technologies and the emergence of geoprocessing, it is possible to collect, display, and process georeferenced information. Statistical methodologies have considered this geographic information system (GIS), incorporating the location of the observation into the analyses. In this study, the occurrence of cases was referred to the municipalities of residence. For the construction of the maps, the cartographic base of Minas Gerais was used through the IBGE website (https://portaldemapas.ibge.gov.br/portal.php#mapa222139). The maps were built in ArcMap v.10.2.2 (https://www.esri.com) and analyzed in the R programming language. GIS allows the database to be connected to geographic features and to the construction of the neighborhood matrix G.

**Figure 1:**
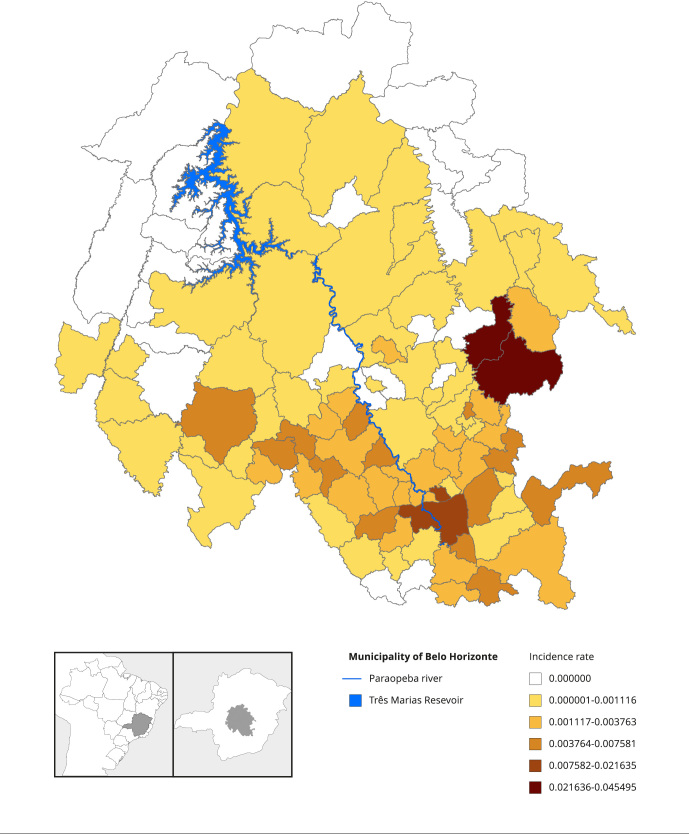
Studied region with the cumulative incidence rate of schistosomiasis, in 103 municipalities in the Minas Gerais State, Brazil, 2007-2020.

Source: elaborated by the authors.

For the spatial analysis, a central region of the State of Minas Gerais, was selected, which includes 103 municipalities around the Paraopeba River basin. Of these, 20 are in direct contact with the bank of the river (proximity 1), 40 are at proximity 2, and 43 at proximity 3. Figure 1 illustrates the studied region, highlighting the municipalities.

The region under study includes municipalities with populations of varying sizes, including the capital of Minas Gerais. The climate of the region is of the subhumid temperate type (the capital is close to 853 meters from sea level). The classification according to Köppen for the region is tropical savannah, bordering humid temperate. In the northern part of the region, the prevailing classification is Aw, and the southern part is Cwa ^31^.

## Results and discussion

Periodically, annual data were observed, from 2007 to 2020, in each of the 103 municipalities investigated. The average rates observed per 100,000 inhabitants (total number of cases) between the years 2007 and 2020 were: 7.134 (458), 24.637 (1,430), 44.15 (1,730), 69.478 (2,526), 43.727 (1,649), 12.384 (595), 9.849 (659), 6.386 (574), 7.535 (594), 5.747 (580), 7.981 (507), 7.995 (584), 10.123 (491), and 3.11 (265), respectively. In this period, the total number of cases was 12,642, with an annual average of 903 cases and an average annual rate of 18.588 per 100,000 inhabitants. The cumulative incidence in the period was 204.494 per 100,000 inhabitants.

Generally, high rates were verified in 2010, 2011, and 2012, followed by a downward trend and stabilization, from 2013 to 2019, and a reduction, in 2020. Such findings may be related to a reduction in the number of tests performed over time. Additionally, the implementation of control and treatment policies may have led to a decrease in positive cases observed over time ^32^. It has been verified that the Southeast Region of the country has been presenting a decrease in cases since 2013, mainly in the State of Minas Gerais, despite it still being the state with the highest number of absolute cases and incidence. This is not the case with the Northeast Region, which showed an increase in the number of cases over this period. Thus, the possible decrease in rates may be due to the alteration of the infection dynamics, observing a different profile for the infected and adaptations of the host to new sites ^33^.

**Figure 2:**
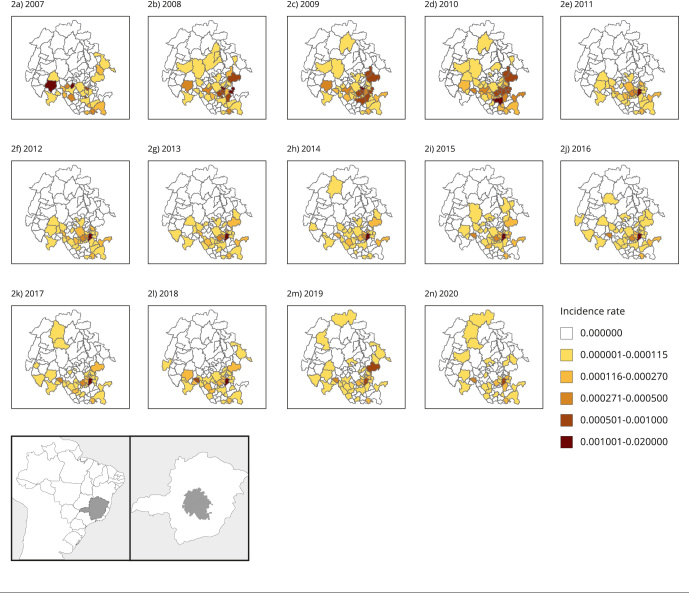
Studied region with the incidence of schistosomiasis per year, in 103 municipalities in the Minas Gerais State, Brazil, 2007-2020.

Source: elaborated by the authors.

Figure 2 shows the spatio-temporal variation with decreased incidence and persistence of the focus in the southern region of the studied area. Although the incidence is decreasing, the endemic region persists.

When evaluating the exogenous variables, we observed a relationship that may be nonlinear with the parameters, with the presence of asymmetry in the distribution and heteroscedasticity of the dependent variable. An important fact to consider is the distribution of data for each municipality. In this case, the variable presents distinct destructions for each municipality, evidencing the need to model the scale and form of the distribution due to the presence of zeros in some municipalities and extreme values in others. These behaviors need to be modeled with appropriateness and with a methodology that can deal with these characteristics. In addition, the mean incidence can be influenced by spatial variation. Spatial correlation test such as Global Moran’s Index and per year were evaluated; a significance (p < 0.05) was found for some of the years evaluated. Another statistic also evaluated was the scan for the presence of spatial and temporal clusters with the SatScan software tool (http://www.satscan.org) ^34^ ^,^ ^35^, being significant for at least 3 spatio-temporal clusters.

Moreover, it was verified that the distribution of incidence behaves differently in space and time, which makes it difficult to choose a methodology that can portray the problem. Several studies have sought to use methodologies that are able to model this type of data in the best way.

Authors such as Wood et al. ^9^ used models with mixed distributions to model the excess of zeros and overdispersion in the data for snail occurrence, in an attempt to determine the spatial-temporal behavior of the species. In the study by Scholte et al. ^36^, the Bayesian geostatistical model was used to predict the disease risk map.

Simões et al. ^8^ also use a methodology that considers spatial variation and Bayesian inference. The authors argue that this methodology allows for the use of a more appropriate distribution for likelihood and priors, with the incorporation of random effects and additional structures for time and space. These studies, despite their different objectives, used different methodologies to deal with the incidence of schistosomiasis. We chose to use GAMLSS models with spatial structure, modeling the temporal effect and seeking the best distribution to deal with the excess of zeros and asymmetry presented by the random variable of interest.

**Figure 3:**
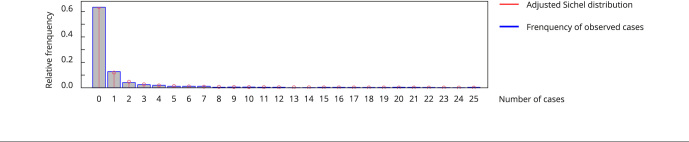
Graphic representation of the frequency of occurrence of schistosomiasis and the adjusted Sichel distribution.

To adjust the number of cases of schistosomiasis, it was necessary to use a Sichel ^37^ mixture distribution to deal with the excess of zeros and long tail. The distribution was selected according to the criteria established by better adjusting the variable of interest. The use of this distribution enabled an adequate representation of the empirical distribution (Figure 3). It is possible to verify the complexity of the modeling due to the presence of more than 60% of the data being zero and a prominent tail on the right. The median of the cases is 0, the third quartile is 1, with a maximum of 477 cases, which characterizes a data set very concentrated in these values and an asymmetric associated distribution. To study the effect of time, the time series of average incidence per year was evaluated, totaling 14 years of registration. We were still able to verify, by the autocorrelation function, the presence of significant *lags*, which characterizes temporal dependence. The Ljung-Box test ^38^ was performed and confirmed significance for *lag* 1 (p = 0.006) and *lag* 2 (p = 0.016). This result justifies the presence of the variables of first and second order lags in the modeling Material Suplementar: https://cadernos.ensp.fiocruz.br/static//arquivo/suppl-e00068822-eng_2815.pdf).

The Sichel distribution is a mixture of the Poisson distribution and the generalized inverse normal. This distribution presents three y~Sichel parameters: mean (*μ*), variability (*σ*), and form (*υ*). When σ tends to infinity, Sichel tends to the Poisson distribution (*μ*); and when σ tends to infinity and *υ*> 0, it tends to negative binomial. Logarithmic link functions for μ and σ and identity for υ ^30^ were used for the analysis.

After defining the distribution that would be used, the adjustment process began by using the model selection strategy presented by De Bastiani et al. ^22^, in which two strategic stages were made, adjustment and subsequent adaptation of the model using normalized randomized quantile residuals ^28^, worm-plot, and Q statistics ^29^, in addition to the evaluation of independence. The selection of variables to compose the final model was performed by stepwise GAIC.

Table 1 presents the final model with all significant variables at 5%. The exogenous variables that are not composing the model were removed since they did not present statistical significance, according to the stepwise criterion ^39^. Table 1 shows the GMRF, which characterizes the spatial smoothing function or random function considering IAR model and weight matrix of the nearest neighbor type, as a GMRF. In the modeling, interactions between variables were not considered and the use of offset allowed us to work with incidence and, therefore, allowing for the estimation of the risk of occurrence of schistosomiasis in a unit of time/municipality.

**Table 1:** GAMLSS model estimates with parametric and nonparametric functions and random spatial effect.

Parameter	Predictive variables	Estimates	Standard errors	Exponential of the estimate	p-value
*µ*	Intercept	-8.8295	0.1282	0.0001	< 0.001
	*Y*_*i-1*_	0.0248	0.0011	1.0251	< 0.001
	h_11_(*Y*_*i-2*_ )	-			
	h_21_(year)	-			
	GMRF(municipality)	-			
	*B. glabrata* = 1	0.4478	0.1333	1.5649	< 0.001
	*B. straminea* = 1	-0.9811	0.1102	0.3749	< 0.001
	Barragem = 1	0.8128	0.1063	2.2542	< 0.001
*σ*	Intercept	0.4228	0.1159	1.5262	< 0.001
	*Y*_*i-1*_	0.0201	0.0018	1.0203	< 0.001
*υ*	Intercept	-1.0182	0.1055	0.3612	< 0.001
	H_13_ (*Y*_*i-1*_ )	-			

GAMLSS: generalized additive models for location, scale and shape; GMRF: Gaussian Markov random field (spatial function); h: p-splines smoothing function. For the year, a cubic spline function with 9g.l was used. The offset (*log*(population_i_)) was also used in the modeling.

The use of variables that depict the regions where the intermediate hosts were found indicated an important relationship for the follow-up of the disease, especially regarding the species *B. glabrata* and *B. straminea*. According to the estimated model, keeping the other variables constant, the presence of *B. glabrata* is related to higher risk, being 1.56 times higher, when compared to municipalities where this species was not found. For *B. straminea*, this represents an inverse relationship to incidence, and its presence has a 62.51% lower risk. It was expected that the presence of *B. glabrata* was actually associated with the occurrence of schistosomiasis ^40^, considering that this is the most important species in the transmission, and it is adapted to the region. The result for the presence of *B. straminea* was interesting. This species is found in several regions of the country because it adapts well to different types of climates ^40^, however, this species is responsible for higher risk of occurrence in the Northeast Region of the country. In Minas Gerais, *B. straminea* is less susceptible than *B. Glabrata* ^10^. Of the area under study, 27.18% of the municipalities have snail species, of which 37.86% confirmed the presence of only *B. glabrata* and 33.98% of only *B. straminea*. The presence of *B. tenagophila* was verified only in 13.59% of the municipalities, and its statistical significance was not observed. This can be explained by the fact that this species is not associated with reports of importance in the transmission of the disease in Minas Gerais, being more present in the Southern Brazil and associated with the disease in the State of São Paulo ^10^.

The three species of *Biomphalaria* have been progressively found in new municipalities, which gives an expansive character to schistosomiasis, including in unaffected areas ^40^, despite positivity being verified in only 1% of the municipalities studied. In the municipality of Belo Horizonte, even after four decades, the three species continue to be found ^7^ in the parks, reinforcing the relationship with the disease. Oliveira et al. ^41^ showed that social inequality is a relevant factor in the incidence of the disease in the State of Minas Gerais. In our study, however, the variables that were associated with social factors, such as M-HDI, sanitation, low income, lack of infrastructure did not present significance. This result can be explained by the region under study, where there is no evident socioeconomic differentiation. Another point that draws attention is that municipalities such as Belo Horizonte and Brumadinho, which presented high numbers of cases, showed satisfactory development indices; therefore, the use of these indicators do not faithfully represent the marginalized population, who are probably more exposed. Another hypothesis to be considered is that these two municipalities may have a more structured epidemiological surveillance system than the others, which could lead to an information bias due to an apparently higher number of cases, but which is, in fact, due to a better notification system and not an actual increase in cases.

Understanding the occurrence of incidence motivated the search for variables that could better characterize the problem. One hypothesis is the possibility of the high incidence in the south of the studied area being associated with the importation of cases, due, for example, to the migration of workers from other regions of Brazil, such as the State of São Paulo and the Northeast Region ^33^. We also observed that a section of the studied area presents a concentration of dams with mining residue. This variable was treated as dichotomous and, according to the model, municipalities with the presence of a dam presented a risk 2.25 times higher than in municipalities without it. This result may indicate a possible migration of positive individuals in these regions due to a greater supply of work and more structured health services.

**Figure 4:**
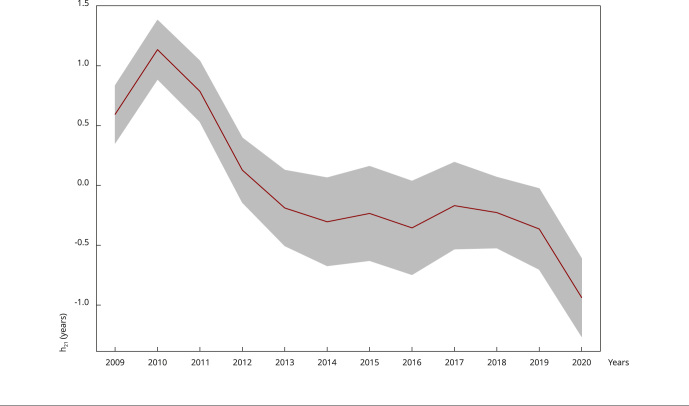
Effects estimated using p-splines smoothing function, associated with the mean parameter with relation to time.

Figure 4 shows the adjustment of the smoothing function for the temporal effect, evidencing the year 2010 as the one with the highest risk, reaching 4 times more risk than the baseline year of 2012. Another point of note is that the year that presented the lowest rate was 2020. Most notably, from 2012 to 2019 the incidences behaved in a constant way, with little spatial variation.

**Figure 5:**
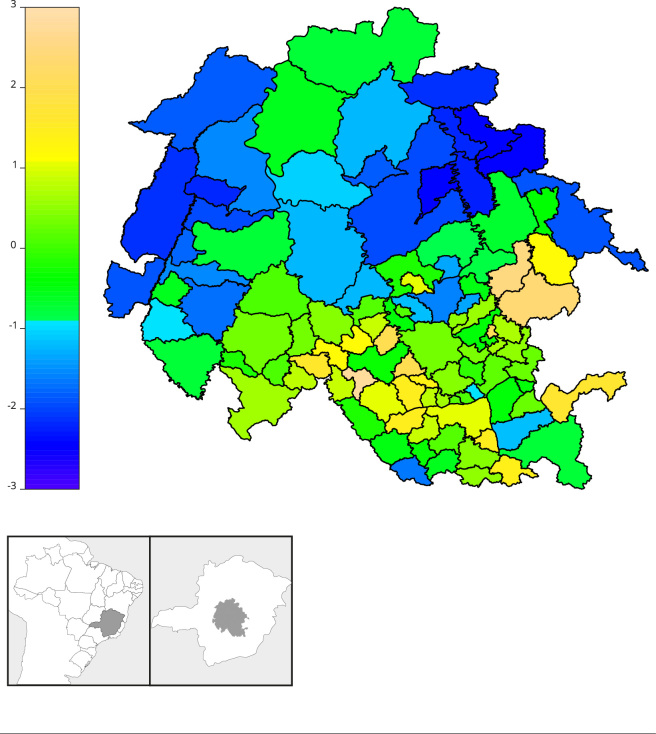
Representation of spatial risk adjusted for the mean parameter. Municipalities of the Minas Gerais State, Brazil, 2007-2020.

The three parameters of the Sichel distribution were modeled with temporal delay variables, which shows that the distribution changes its shape and variability over time, presenting a dependence with the previous period (year). The spatial dependence was verified only in the mean parameter. Figure 5 shows the adjusted spatial effect, the lighter colors characterize a higher risk (exp(*μ*)); thus allowing us to observe regions further south and east of the studied area that presented higher risk in relation to the northern region, possibly reaching over 20 times higher, in some municipalities, when compared to the basal (municipality with value 0 on the map scale).

Nevertheless, we must consider the presence of informational bias due to the underreporting of cases, and due to the study’s ecological approach, at the municipal level, which makes important details unfeasible in a possibly heterogeneous society.

## Conclusions

The GAMLSS model enabled the treatment and modeling of spatio-temporal data, with the use of a Gaussian Markov random field for the treatment of area data, using structured spatial effect, from a contiguity neighborhood matrix. For the temporal adjustment, dependence of orders 1 and 2 and cubic spline function were considered to model the trend.

Our study showed that the control and follow-up of *B. glabrata* snails may be fundamental for the control of schistosomiasis in the studied area. The presence of *B. straminea* snails was inversely associated with the incidence of schistosomiasis in the studied area, and the presence of *B. tenagophila* was not relevant. Another point that deserves a more detailed analysis is the relationship of municipalities with the presence of mining dams and the possible migration of positive individuals, which would need to be better investigated.
