# Supplementary material for: Spatio-temporal GAMLSS modeling of the incidence of schistosomiasis in the central region of the State of Minas Gerais, Brazil
Source: Cad Saude Publica. 2023 Jun 26;39(6):e00068822. doi: 10.1590/0102-311XEN068822 (PMC10494687; doi:10.1590/0102-311XEN068822)
Supplement: Supplementary file 1 [file 1678-4464-csp-39-06-PT068822-s.pdf]

Supplementary material

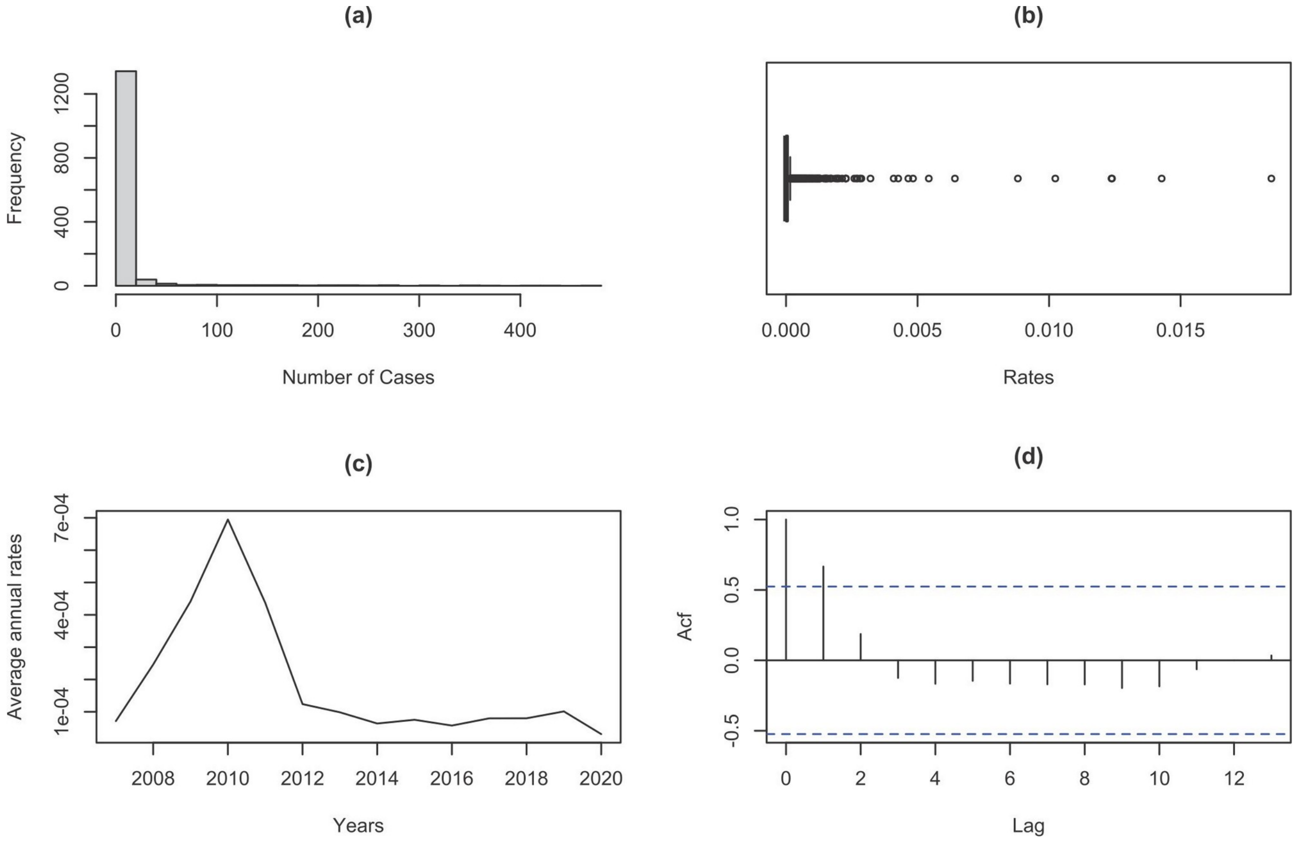

Figure S1 Graphical representation of the frequency of occurrence of schistosomiasis using the bar graph (a), the distribution using the boxplot (b), the time series of the mean incidence per year (c) and the incidence autocorrelation function (d).
